# Transition metal ions regulated oxygen evolution reaction performance of Ni-based hydroxides hierarchical nanoarrays

**DOI:** 10.1038/srep46154

**Published:** 2017-04-06

**Authors:** Tingting Zhou, Zhen Cao, Pan Zhang, Houyi Ma, Zhen Gao, Heng Wang, Yue Lu, Jia He, Yunfeng Zhao

**Affiliations:** 1 School of Chemistry and Chemical Engineering, Shandong University, Jinan 250100, China; 2 Tianjin Key Laboratory of Advanced Functional Porous Materials, Institute for New Energy Materials and Low-Carbon Technologies, Tianjin University of Technology, Tianjin 300384, China

## Abstract

Nickel-based hydroxide hierarchical nanoarrays (Ni_*y*_M(OH)_x_ HNAs M = Fe or Zn) are doped with non-noble transition metals to create nanostructures and regulate their activities for the oxygen evolution reaction. Catalytic performance in these materials depends on their chemical composition and the presence of nanostructures. These novel hierarchical nanostructures contain small secondary nanosheets that are grown on the primary nanowire arrays, providing a higher surface area and more efficient mass transport for electrochemical reactions. The activities of the Ni_*y*_M(OH)_x_ HNAs for the oxygen evolution reaction (OER) followed the order of Ni_2.2_Fe(OH)_*x*_ > Ni(OH)_2_ > Ni_2.1_Zn(OH)_*x*_, and these trends are supported by density functional theory (DFT) calculations. The Fe-doped nickel hydroxide hierarchical nanoarrays (Ni_2.2_Fe(OH)_*x*_ HNAs), which had an appropriate elemental composition and hierarchical nanostructures, achieve the lowest onset overpotential of 234 mV and the smallest Tafel slope of 64.3 mV dec^−1^. The specific activity, which is normalized to the Brunauer–Emmett–Teller (BET) surface area of the catalyst, of the Ni_2.2_Fe(OH)_*x*_ HNAs is 1.15 mA cm^−2^_BET_ at an overpotential of 350 mV. This is ~4-times higher than that of Ni(OH)_2_. These values are also superior to those of a commercial IrO_x_ electrocatalyst.

Because of growing demands for energy and environmental concerns, advanced technologies are being sought for the production of inexpensive, sustainable, and carbon-neutral fuels^1^,^2^,^3^,^4^,^5^. Water splitting is promising for the production of fuel from renewable but intermittent energy sources, such as wind and solar^6^. The anodic water-splitting reaction, the oxygen evolution reaction (OER), is a significant source of efficiency losses, because it involves multistep proton-coupled electron transfer and sluggish kinetics^7^. Currently, noble metal-based compounds, such as IrO_x_ and RuO_2_, are the most active OER catalysts, but their scarcity and high cost limit their use in practical applications^8^,^9^,^10^,^11^. Therefore, alternative electrode configurations are needed with extraordinary activities and superior long-term stabilities for the OER^12^,^13^,^14^.

Low-cost transition metal catalysts, especially Ni^15^,^16^, have been studied extensively, exhibit good catalytic activities, and are stable against corrosion during the OER in alkaline media. The development of improved Ni-based OER catalysts can be accelerated by an improved understanding of the intrinsic catalytic activity of materials and their dependence on catalyst composition and structure. The OER activity of Ni-based catalysts can be significantly enhanced by doping with other earth-abundant elements such as Co, Fe, Mn, and Zn^17^,^18^,^19^,^20^,^21^,^22^. Therefore, a systematic study of the OER activities achieved when different dopants are added to Ni-based hydroxides would provide valuable insight into the synthesis of improved OER catalysts^23^. Recently, experimental studies suggest that the OER activities of oxyhydroxide thin films follow the order of Ni(Fe)O_*x*_H_*y*_ > Co(Fe)O_*x*_H_*y*_ > FeO_*x*_H_*y*_-AuO_*x*_ > FeO_*x*_H_*y*_ > CoO_*x*_H_*y*_ > NiO_*x*_H_*y*_ > MnO_*x*_H_*y*_^24^. Experimental studies have shown that the overpotentials required to achieve 10 mA cm^−2^ for heterogeneous electrocatalysts follow the order of NiFeO_*x*_ < CoFeO_*x*_ < NiCoO_*x*_ CoO_*x*_ < NiLaO_*x*_ < NiCuO_*x*_ < CoO_*x*_/CoPi < NiO_*x*_ < NiCeO_*x*_^25^. However, the performance of OER catalysts can be limited by the low conductivities^24^ and surface areas^25^ of ordinary nanostructures, particularly those containing nanoparticles. Previous studies have shown that the OER activity of an ultra-thin γ-CoOOH nanosheet is 20-times higher than that of bulk CoOOH and 2.4-times higher than that of an IrO_2_ electrocatalyst^26^. Well-aligned nanowire arrays have been used as highly effective electrodes, because of their intrinsic advantages^27^,^28^,^29^. Moreover, ordinary electrodes have relatively poor stabilities because of contact between the substrate and electrocatalysts during the electrocatalytic of OER, especially at large current densities. Therefore, hierarchically architectures can be constructed on conductive metal substrates to form high-performance nanocatalysts electrode^30^,^31^. However, no systematic studies have been performed that combine theoretical and experimental characterizations of the relationship between the doping of transition metals and the OER activities of Ni-based hydroxide nanoarrays.

In this work, the nanostructured morphology and OER activity of Ni-based hydroxide hierarchical nanoarrays (Ni_*y*_M(OH)_x_ HNAs, M = Fe or Zn) were modified using two non-noble transition metals (Fe and Zn) as dopants. A systematic experimental and theoretical study of the effect of transition-metal doping on the nanostructure and OER activity of nickel-based catalysts is presented in this work. The intrinsic OER activity trends of the Ni_*y*_M(OH)_x_ HNAs followed Ni_2.2_Fe(OH)_*x*_ > Ni(OH)_2_ > Ni_2.1_Zn(OH)_*x*_. Theoretical and experimental results were in good agreement. The trends were explained in terms of the surface areas and compositions of active sites, providing potential insights for the future design of more efficient water-splitting catalysts.

## Results and Discussion

**Figure 1:**
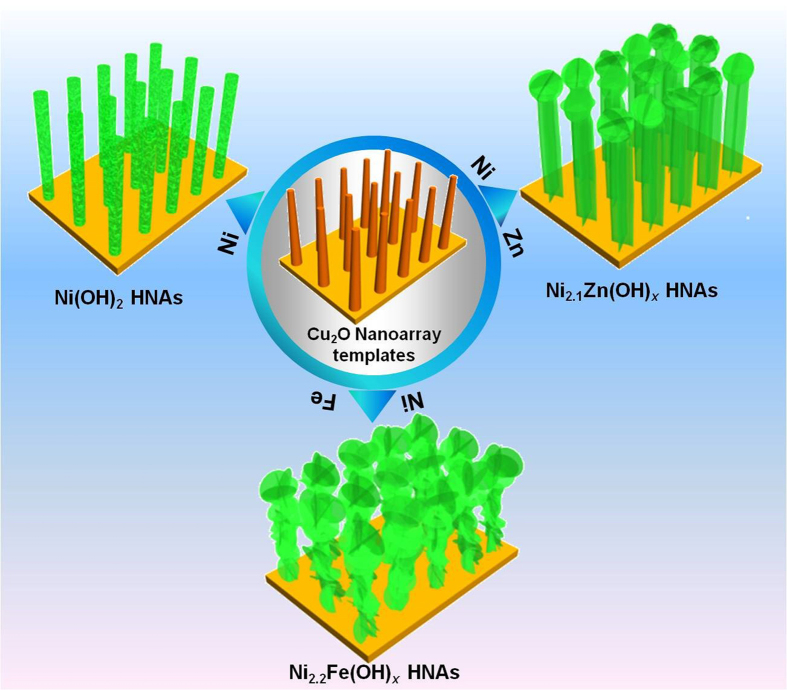
Depiction of the growth of Zn- and Fe-doped Ni_*y*_M(OH)_*x*_ HNAs from sacrificial Cu_2_O nanoarray templates.

**Figure 2:**
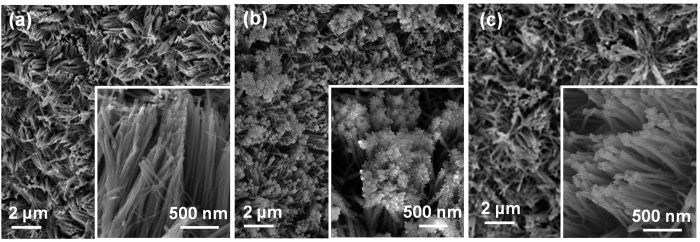
Low- and high-magnification (inset) SEM images of the (**a**) Ni(OH)_2_, (**b**) Ni_2.2_Fe(OH)_*x*_, and (**c**) Ni_2.1_Zn(OH)_*x*_ HNAs.

**Figure 3:**
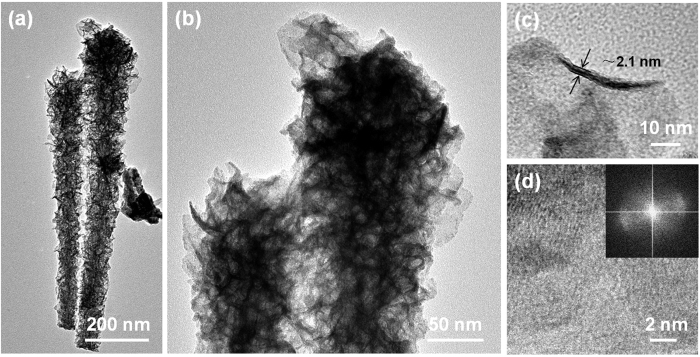
(**a**,**b**) TEM images, (**c**) high-magnification TEM image, and (**d**) HRTEM and FFT (inset) images of the Ni_2.2_Fe(OH)_*x*_ HNAs.

Ni_*y*_M(OH)_x_ HNAs, where M = Fe and Zn, were fabricated by dipping Cu foam substrates coated with one-dimensional (1D) Cu_2_O nanowire arrays into an aqueous solution containing metal chloride salts and sodium hyposulfite using a solution-phase cation exchange method at room temperature. During the cation exchange process, the Cu_2_O nanowires were etched by S_2_O_3_^2−^, releasing OH^−^. During this process Ni_*y*_M(OH)_*x*_ HNAs precipitated, these new Ni_*y*_M(OH)_*x*_ HNAs structures inherited the geometry of the Cu_2_O template. Secondary Ni_*y*_M(OH)_*x*_ HNAs nanostructures also formed depending on the solubility of the products and the pH of the reaction system. As illustrated in Fig. 1, the secondary nanostructures of the Ni_*y*_M(OH)_x_ HNAs were regulated during this process. Low magnification SEM images of the Ni_*y*_M(OH)_*x*_ HNAs revealed that the surface of the Cu foam substrate was completely covered with vertically aligned nanoarrays (Fig. 2). The inset to Fig. 2a showed the morphology of the Ni(OH)_2_ HNAs, which inherited the shape of the 1D Cu_2_O nanowire arrays (Fig. S1) along the axial direction. After doping Ni(OH)_2_ with transition metals, the surfaces of the nanowires became rougher, and their morphologies markedly changed into hierarchical structures with secondary nanosheets grown on the primary nanowire arrays (see insets to Fig. 2b,c). The degree of surface roughness on the Ni_*y*_M(OH)_*x*_ HNAs followed the order of Ni(OH)_2_ < Ni_2.1_Zn(OH)_*x*_ < Ni_2.2_Fe(OH)_*x*_, indicating a marked increasement in surface area when the appropriate elements were used as dopants. Transmission electron microscopy (TEM) images of the Ni_*y*_M(OH)_*x*_ HNAs further revealed the presence of secondary nanosheets (Figs 3 and S2). The Ni_2.2_Fe(OH)_*x*_ HNAs had the most irregularly shaped nanosheet coating. As shown in Fig. S3, the Ni(OH)_2_, Ni_2.1_Zn(OH)_*x*_, and Ni_2.2_Fe(OH)_*x*_ HNAs had diameters of 30.5 nm, 51 nm, and 103.4 nm, respectively. Cross-sectional SEM images of the Ni_*y*_M(OH)_*x*_ HNAs (Fig. S4) revealed that the three Ni_*y*_M(OH)_*x*_ HNAs had similar lengths of 2 μm. Figure 3c,d show high-magnification and high-resolution TEM (HRTEM) images of the Ni_2.2_Fe(OH)_*x*_ HNAs. Ultra-thin (~2.1 nm) character was clearly observed on one edge curled nanosheet of the Ni_2.2_Fe(OH)_*x*_ HNAs, indicating more exposure of low coordinated surface atoms and thus abundant catalytically active sites. HRTEM images indicated that the Ni(OH)_2_ HNAs were predominantly crystalline, while the Ni_2.2_Fe(OH)_*x*_ and Ni_2.1_Zn(OH)_*x*_ HNAs were amorphous (Figs S2b, 3d and S2e). Fast Fourier transform (FFT) images were in agreement with the HRTEM images. SEM and TEM results indicated that the proposed method effectively regulated the growth of nanostructures on the Ni(OH)_2_ HNAs using Fe and Zn as dopants. When doping with the transition metals Fe and Zn, the surfaces of the HNAs become rougher and more highly amorphous. Figure S5 shows X-ray diffraction (XRD) patterns for the Cu_2_O nanowire arrays and the Co_*y*_Fe_*1-y*_(OH)_*x*_ HNAs. The diffraction patterns for the Cu_2_O nanowire arrays indicated the presence of Cu_2_O phases (PDF#65-3288) and Cu (PDF# 65-9743). The diffraction patterns for the Ni(OH)_2_, Ni_2.1_Zn(OH)_*x*_, and Ni_2.2_Fe(OH)_*x*_ HNAs did not contain any characteristic peaks for Ni, Fe, or Zn compounds. Only Cu and a small amount of Cu_2_O were present (Fig. S5), revealing that the three samples had amorphous structures. It should be noted that the amorphous nature of the Ni(OH)_2_ HNAs observed by XRD did not conflict with the crystal structures obtained from HRTEM, because the faint crystal lattice and weak FFT pattern of the Ni(OH)_2_ HNAs indicated a low crystallinity^32^,^33^.

**Figure 4:**
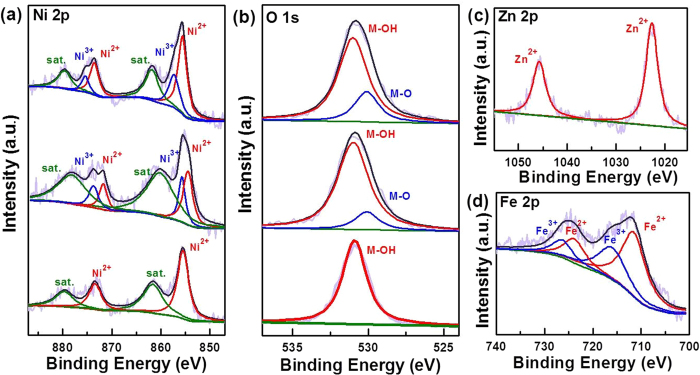
High-resolution XPS spectra of the (**a**) Ni 2p and (**b**) O 1s regions for the Ni(OH)_2_, Ni_2.1_Zn(OH)_*x*_, and Ni_2.2_Fe(OH)_*x*_ HNAs (from bottom to top); (**c**) Zn 2p spectra of the Ni_2.1_Zn(OH)_*x*_ HNAs, (**d**) Fe 2p spectra of the Ni_2.2_Fe(OH)_*x*_ HNAs.

The Ni/M atomic ratios of the Ni_*y*_M(OH)_*x*_ HNAs were determined with inductively coupled plasma (ICP) emission spectrometry. The ratios in the HNAs were similar to the reactant ratios (Table S1), indicating that the Ni/M ratios in the hydroxides were similar to those in the precursors. The surface compositions and valence states of the as-prepared Ni_*y*_M(OH)_*x*_ HNAs were investigated by X-ray photoelectron spectroscopy (XPS), and the results are shown in Fig. 4. Nickel, Fe, Zn, and O species were observed. Peak fitting analysis of the Ni 2p for Ni(OH)_2_ revealed one Ni^2+^ state for Ni at binding energies of 855.5 eV and 873.5 eV. Peak fitting analysis for Ni 2p in the Ni_2.2_Fe(OH)_*x*_ and Ni_2.1_Zn(OH)_*x*_ HNAs indicated the presence of Ni^2+^ (855.4 eV and 873.1 eV) and Ni^3+^ (857.3 eV and 875.5 eV)^34^. Compared to the un-doped Ni(OH)_2_ HNAs, the Ni 2p peaks of the M-doped samples were shifted to more positive energies (Ni_2.1_Zn(OH)_*x*_ < Ni_2.2_Fe(OH)_*x*_), suggesting that the oxidation of Ni^2+^ was favored when Fe and Zn were added. This effect was strongest with Fe^35^. Additional evidence for the presence of Ni^2+^ was observed in the two intense shakeup satellite peaks (861.8 eV and 880.0 eV)^16^. The Zn 2p XPS spectrum for the Ni_2.1_Zn(OH)_*x*_ HNAs contained 2p_3/2_ and 2p_1/2_ doublets, which are characteristic of Zn^2+^ (1022.7 eV and 1045.7 eV)^34^. Fe 2p_3/2_ and Fe 2p_1/2_ spin-orbital splitting for the Ni_2.2_Fe(OH)_*x*_ HNAs was deconvolved into four peaks, indicating the coexistence of Fe^2+^ (711.5 eV and 723.7 eV) and Fe^3+^ (716.0 eV and 726.3 eV) in the Ni_2.2_Fe(OH)_*x*_ HNAs^36^,^37^. The O 1s spectrum of the Ni(OH)_2_ HNAs was fit to a peak at a binding energy of 530.9 eV, which was assigned to the oxygen in hydroxide. The O 1s spectra of the Ni_2.2_Fe(OH)_*x*_ and Ni_2.1_Zn(OH)_*x*_ HNAs were fit with two peaks at binding energies of 530.1 eV and 531 eV, revealing the presence of lattice and hydroxide oxygens, respectively^16^. These results confirm that strong electron interactions occurred between Ni and both Fe and Zn in the Ni_*y*_M(OH)_*x*_ HNAs.

**Figure 5:**
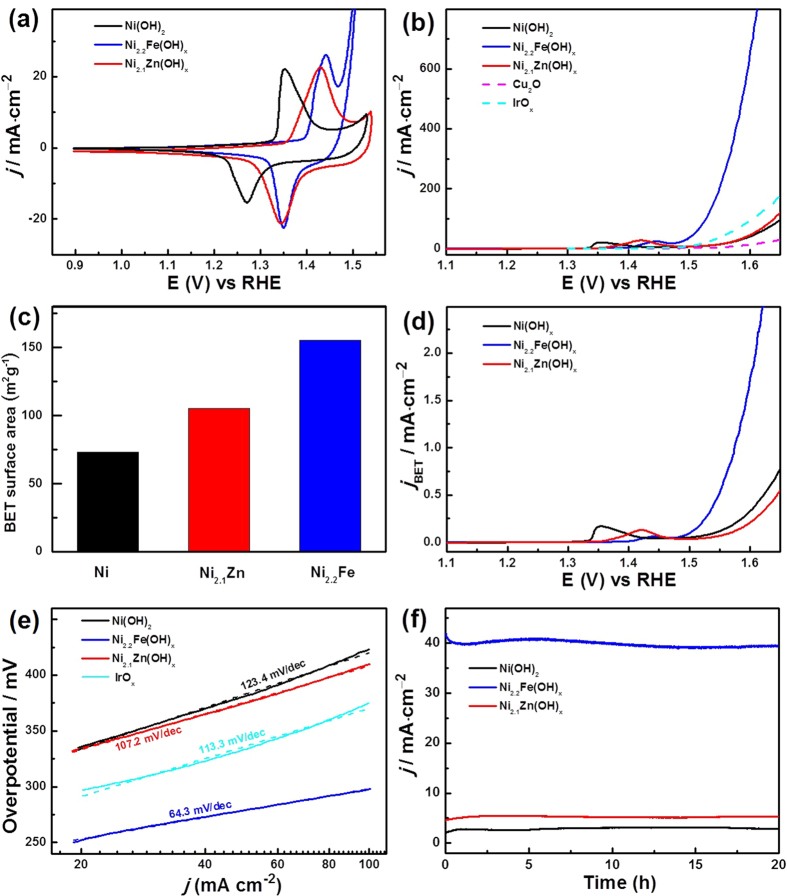
(**a**) CV curves of the Ni_*y*_M(OH)_*x*_ HNAs. (**b**) LSV curves of the Ni_*y*_M(OH)_*x*_ HNAs, Cu_2_O nanoarray, and IrO_x_. (**c**) BET surface areas (**d**) LSV curves normalized to the BET surface areas of the Ni_*y*_M(OH)_*x*_ HNAs. (**e**) Tafel plots for the Ni_*y*_M(OH)_*x*_ HNAs and IrO_x_. (**f**) Chronoamperometric measurements for the Ni_*y*_M(OH)_*x*_ HNAs.

The effect of doping the Ni_*y*_M(OH)_*x*_ HNAs on their electrochemical behaviors were investigated using cyclic voltammetry (CV) in 1 M KOH. As shown in Fig. 5a, the CV curves of all three electrodes exhibited coupled redox peaks. The redox couple (1.45 V/1.27 V *vs*. RHE) of the Ni(OH)_2_ HNAs corresponded to the transformation between Ni(OH)_2_ and NiOOH^16^. The Ni_2.1_Zn(OH)_*x*_ and Ni_2.2_Fe(OH)_*x*_ HNAs’ exhibited redox peaks at 1.43 V/1.34 V *vs*. RHE and 1.44 V/1.35 V *vs*. RHE, respectively. These peaks corresponded to the transformation between Ni(OH)_2_ and NiOOH, and the positive shifts in redox potential were caused by adding the dopants (Fe or Zn) to the electrodes^19^,^38^.

The effect of doping the Ni_*y*_M(OH)_*x*_ HNAs on their OER catalytic activities were tested with linear sweep voltammetry (LSV) in 1 M KOH (Fig. 5b). The Cu_2_O nanoarrays exhibited a negligible catalytic activity, while the OER current of the Ni_2.2_Fe(OH)_*x*_ HNAs was much higher than those of the other electrodes. The Ni_2.2_Fe(OH)_*x*_ HNAs had a low OER onset overpotential (η) of 234 mV, which was more negative than the η of the Ni(OH)_2_ HNAs (254 mV) and commercial IrO_x_ electrocatalyst (248 mV). The high catalytic activity of the Ni_2.2_Fe(OH)_*x*_ HNAs was also indicated by its ability to support a given current density (*j*) at a lower η than the other electrodes. At *j* = 100 mA cm^−2^, the as-prepared Ni_2.2_Fe(OH)_*x*_ HNAs required an η of 298 mV, which was 125 mV and 177 mV less than the η values of the Ni(OH)_2_ HNAs (423 mV) and IrO_x_ (375 mV), respectively. Therefore, intrinsic activities were compared at η = 350 mV. The intrinsic activity of the Ni_2.2_Fe(OH)_*x*_ HNAs was 16- and 5-times higher than those of the Ni(OH)_2_ HNAs and IrO_x_, respectively, revealing strong interactions between Ni and Fe during OER catalysis. Meanwhile, the Ni_2.1_Zn(OH)_*x*_ HNAs exhibits a more positive onset η of 276 mV, and required a high η of 410 mV to achieve a current density of 100 mA cm^−2^. The intrinsic activity of the Ni_2.1_Zn(OH)_*x*_ HNAs (25.8 mA cm^−2^) was similar to that of the Ni(OH)_2_ HNAs. These results suggest that unfavorable interactions occurred between Ni and Zn. The excellent OER activities of the catalysts were attributed to their increased surface areas and specific activities (active sites per unit area).

The Brunauer–Emmett–Teller (BET) surface area measurements were performed to confirm the mesoporous nature of the Ni_*y*_M(OH)_*x*_ HNAs. Nitrogen adsorption-desorption curves revealed a Type IV isotherm (Fig. S8). Additionally, a H3-type hysteresis loop was observed, providing further evidence of nanosheet aggregation^39^. As shown in Fig. 5c, the Ni(OH)_2_ HNAs (73.2 cm^2^ g^−1^) had a smaller BET surface area than the Ni_2.1_Zn(OH)_*x*_ (105.6 cm^2^ g^−1^) and Ni_2.2_Fe(OH)_*x*_ (155.6 cm^2^ g^−1^) HNAs. These results confirmed observations of increased surface areas in the SEM (Fig. 2) and TEM (Figs 3 and S2) images. Specific activity (current per BET area) is a measure of the density of active sites on the surface of a catalyst. Figure 5d shows LSV curves after normalizing the measured currents to the catalysts’ BET surface areas. The specific activity of the Ni_2.2_Fe(OH)_*x*_ HNAs was 1.15 mA cm^−2^_BET_ at η = 350 mV, which was 4- and 8-times higher than those of the Ni(OH)_2_ HNAs (0.22 mA cm^−2^_BET_) and Ni_2.1_Zn(OH)_*x*_ HNAs (0.13 mA cm^−2^_BET_), respectively. These results indicated that adding Fe indeed resulted in more active sites, while doping with Zn decreased the number of active sites.

Kinetic analyses were performed using LSV to generate Tafel plots and electrochemical impedance spectra (EIS). As shown in Fig. 5e, the resulting Tafel slope of the Ni_2.2_Fe(OH)_*x*_ HNAs was 64.3 mV dec^−1^, which was much lower than that of the Ni(OH)_2_ HNAs (123.4 mV dec^−1^), Ni_2.1_Zn(OH)_*x*_ HNAs (107.2 mV dec^−1^), and IrO_x_ (113.3 mV dec^−1^). Tafel slopes were used to probe the OER mechanisms of the catalysts. Efficient electron and mass transport result in lower Tafel slopes. The EIS was performed in oxygen-saturated 1.0 M KOH (Fig. S6). ZSimpWin 3.5 (Zolartron Analytical) was used to fit the resistance values, as shown in Table S2. As shown in the inset to Fig. S6, all of the Ni_*y*_M(OH)_*x*_ HNAs electrodes were fitted using the same equivalent circuit, which contained three components: solution resistance (R_s_), charge-transfer resistance (R_ct_), and constant-phase resistance (R_cp_). The Ni_2.2_Fe(OH)_*x*_ HNAs had an R_ct_ of 1.7 Ω, which was much lower than that of the Ni_2.1_Zn(OH)_*x*_ (21.4 Ω) and Ni(OH)_2_ (23.8 Ω) HNAs. These results indicated that OER kinetics were enhanced for the Ni_2.2_Fe(OH)_*x*_ HNAs electrode. These EIS measurements were consistent with the findings from LSV.

Turnover frequency (TOF) is an intrinsic property of a catalyst and an important indicator of catalyst performance. The TOF of the Ni_2.2_Fe(OH)_*x*_ HNAs was much higher than that of Ni_2.1_Zn(OH)_*x*_ and Ni(OH)_2_ HNAs (Fig. S7). Moreover, at η = 350 mV, the TOF of the Ni_2.2_Fe(OH)_*x*_ HNAs was at least 15-, 21-, and 3-times as those of the Ni(OH)_2_ HNAs (0.011 s^–1^), the Ni_2.1_Zn(OH)_*x*_ HNAs (0.008 s^–1^), and IrO_x_ (0.05 s^–1^), respectively. These TOF values further verified the superior catalytic performance of the Ni_2.2_Fe(OH)_*x*_ HNAs for the OER. This improved performance resulted from strong interactions between Ni and Fe and the presence of more exposed catalytically active sites.

Long-term stability is also important for catalysts that are to be used for practical applications. As shown in Fig. 5f, all of the Ni_*y*_M(OH)_*x*_ HNAs possessed excellent stabilities with nearly no decrease in *j* after over 20 h of operation in O_2_-saturated 1 M KOH. This remarkable operational stability was ascribed to the efficiency of the current collector, the material’s excellent intrinsic stability, the robustness of the electrode and a low coverage of gas bubbles on the Ni_*y*_M(OH)_*x*_ HNAs^40^. The higher *j* values achieved by the Ni_2.2_Fe(OH)_*x*_ HNAs was attributed to the presence of hierarchical nanostructures and strong interactions between Ni and Fe. The superior long-term stability of the Ni_2.2_Fe(OH)_*x*_ HNAs suggests its potential use as a new electrode in oxygen-evolution devices. The electrochemical properties of Ni_*y*_M(OH)_*x*_ and IrO_*x*_ are summarized in Table 1, and especially the overall performance of Ni_2.2_Fe(OH)_*x*_ surpasses most reported typical Co-based electrocatalysts for water oxidation under alkaline solution (Table S3).

**Figure 6:**
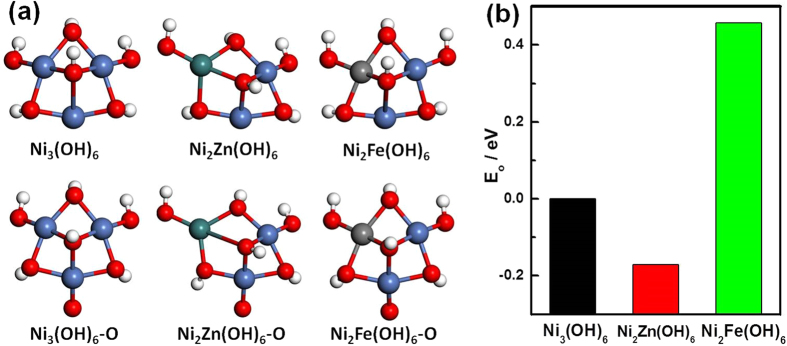
(**a**) Optimal hydroxide models and adsorption models. Atoms in the clusters: blue = Ni; green = Zn; gray = Fe; red = O; white = H. (**b**) E_o_ on the different hydroxide clusters.

To better understand the catalytic activities that resulted from doping with Fe and Zn, the binding energies of oxygen on the Ni_*y*_M(OH)_*x*_ catalysts were investigated using DFT calculations. The binding energy of oxygen serves as a reliable measure of the activity of a catalyst toward the OER. Smaller values of binding energy of oxygen(E_o_) at a reaction site correspond to higher activities for the OER^41^,^42^. Hydroxide clusters were constructed based on a model of Ni_2_M(OH)_6_. The oxygen binding energies of the hydroxide clusters increased in the order of Ni_2_Zn(OH)_6_ > Ni_3_(OH)_6_ > Ni_2_Fe(OH)_6_ (Fig. 6b). The reactivities of the amorphous hydroxides followed the order of Ni_2_Fe(OH)_6_ > Ni_3_(OH)_6_ > Ni_2_Zn(OH)_6_, which was in good agreement with the experimental results.

## Conclusion

Nanostructures were generated to regulate the OER activities of the Ni_*y*_M(OH)_x_ HNAs using various non-noble transition metals as dopants. According to both experimental and DFT-based theoretical analyses, Fe and Zn had opposite effects on the catalyst’s activity. Fe was an effective dopant, while Zn decreased the OER activity of the catalyst. Hierarchical nanostructures allowed efficient charge transfer and a sufficient surface area for active sites. A 3D porous Cu foam not only provided a large surface area and stable anchoring sites for nanoarrays but also acted as an efficient electron collector. Because of the hierarchical nanostructures, the appropriate elemental composition of the catalyst, and the presence of a multifunctional 3D conductive substrate, the Ni_2.2_Fe(OH)_*x*_ HNAs exhibited an enhanced OER activity. The Ni_2.2_Fe(OH)_*x*_ HNAs had a low onset η of 234 mV and a small Tafel slope of 64.3 mV dec^−1^. They also exhibited an excellent long-term stability for over 20 h in an alkaline electrolyte. The Ni_2.2_Fe(OH)_*x*_ HNAs also had a superior activity compared to that of a commercial IrO_*x*_ catalyst, and these Ni_2.2_Fe(OH)_*x*_ HNAs were prepared using an extremely simple method. Their activities were comparable to those of other NiFe hydroxides obtained through more labor-intensive procedures. This study provides significant new guidelines for and a broader understanding of the use of dopants to improve catalytic activity.

## Methods

### Materials synthesis

Nickel(II) dichloride (NiCl_2_·6H_2_O, AR), iron (II) dichloride (FeCl_2_∙4H_2_O, AR), zinc dichloride (ZnCl_2_, AR), potassium hydroxide (KOH, AR), sodium hyposulfite (Na_2_S_2_O_3_∙5H_2_O, AR), oxalic acid (H_2_C_2_O_4_, AR), and ethanol (CH_3_CH_2_OH, AR) were purchased from Sinopharm Chemical Reagent Co., Ltd. All chemicals were used as received without further purification. Mill-Q water (resistivity > 18 MΩ·cm) was used throughout.

Cu foam (100 pores per inch, 98% porosity, and ~1.5 mm thick) was cut into squares (2.0 cm × 2.0 cm), and cleaned in Mill-Q water and ethanol before use. The Cu foam was then anodized in 0.4 M H_2_C_2_O_4_ for 20 min at 36 V, using a graphite plate cathode. Electro-oxidation was performed with a potentiostat (CHI760D, CH Instruments) in a three-electrode configuration consisting of an anodized Cu foam working electrode, a Pt gauze counter electrode, and an Ag/AgCl reference electrode^43^. Cyclic voltammetry was performed in the potential range from −0.3 V to 0.1 V at a scan rate of 1 mV s^−1^ in 1 M KOH for the *in situ* growth of Cu_2_O nanowire arrays on the Cu foam^43^,^44^.

Ni_*y*_M(OH)_x_ HNAs were fabricated using the Cu_2_O nanowire arrays as sacrificial templates^33^. Briefly, known amounts of NiCl_2_·6 H_2_O and MCl_2_·nH_2_O (M = Fe or Zn) were dissolved in a mixture containing 17.5 mL Mill-Q water and 17.5 mL ethanol. A 2:1 molar ratio of NiCl_2_·6H_2_O and MCl_2_·nH_2_O was used to prepare the Ni_*y*_M(OH)_x_ HNAs. The total moles of NiCl_2_·6H_2_O and MCl_2_·nH_2_O used was 8 × 10^−5^. A Cu foam decorated with Cu_2_O nanowire arrays was immersed in this suspension, which was then stirred at room temperature. Na_2_S_2_O_3_ (1 M, 10 mL) was added dropwise to this mixture under magnetic stirring for 1 h. The substrate was then removed and washed repeatedly in ethanol and Mill-Q water before being dried at 60 °C in a vacuum oven for 4 h.

### Structural characterization

Scanning electron microscopy (SEM) was performed with a ZEISS MERLIN scanning electron microscope. Microstructural investigations were performed with a JEOL JEM-2100 and Tecnai G2 Spirit TWIN. X-ray diffraction (XRD) patterns were recorded using a Rigaku Ultima IV. The valence states of elements were measured with X-ray photoelectron spectroscopy (XPS, PHI 5000 VersaProbe). All of the spectra were normalized to the C 1 s binding energy at 284.8 eV. Ni/M atomic ratios were measured with a VISTA-MPX ICP-OES. BET measurements were performed on a Quadrasorb SI analyzer at 77 K.

### Electrochemical Measurements

Electrochemical measurements were performed in O_2_-saturated 1 M KOH with an electrochemical analyzer (CHI760D, CH Instruments), using a three-electrode configuration with an Hg/HgO (1 M KOH) reference electrode that contained a double salt bridge and a platinum mesh counter electrode. The Ni_*y*_Fe_*1-y*_(OH)_*x*_ HNAs (0.5 cm × 0.5 cm) on Cu foams were used as working electrodes. All polarization measurements were performed at a scan rate of 5 mV s^−1^. Potentials are reported in terms of the reversible hydrogen electrode (RHE), using: *E* (RHE) = *E* (Hg/HgO) + 0.098 V + 0.0591 V × pH. All CV measurements were compensated for *iR* drop by 75%. Stability was measured using the controlled potential electrolysis method. The EIS was performed with a Princeton PMC 1000 electrochemical workstation in the frequency range of 10^−2^ Hz−10^4^ Hz at an amplitude of 5 mV. All electrochemical tests were performed at 25 °C.

Turnover frequency (TOF) was calculated as: TOF = (*j* × *a*)/(4 × *n* × *F*), where *j* is the current density at a given potential, *a* is the surface area of the electrode (0.25 cm^2^ for the Cu foam electrode), 4 is the number of electrons transferred in the OER, *n* is the number of moles of all metal ions available for the OER (including Ni and M), and *F* is Faraday’s constant (96485 C mol^−1^).

### DFT Calculations

Since the Ni_*y*_M(OH)_x_ HNAs were predominantly amorphous (the Ni(OH)_2_ HNAs had a hexagonal Ni(OH)_2_ phase and the Ni_2_M(OH)_*x*_ HNAs had an amorphous phase), cluster rather than slab model was chosen for the DFT simulation. Hydroxide clusters were first built based on the model of Ni_2_M(OH)_6_^45^, as shown in Fig. 6a. All reported DFT calculations with the Hubbard U (DFT + U) calculations were performed at the Perdew-Burke-Ernzenhof/Generalized Gradient Approximation (PBE/GGA)^46^ level using the spin-dependent formulation of the hybrid Gaussian and the plane waves method. The calculations were implemented with the open-source CP2K/QUICKSTEP^47^,^48^ code. For a better description of the Ni and Fe 3d electrons, the Hubbard effective terms U_eff_(Ni) = 5.96 eV and U_eff_(Fe) = 5.3 eV were added to the PBE functional^49^,^50^. Electrons in the outer most shells of the atoms were treated as being in their valence states. The Kohn-Sham orbitals of the valence electrons were expanded in molecularly optimized Gaussian basis sets of double-ζ plus polarization quality (MOLOPT-SR-DZVP)^51^. Ionic cores were represented by norm-conserving Goedecker-Teter-Hutter^52^,^53^,^54^ pseudopotentials. The auxiliary plane wave basis set was truncated with a 500 Ry kinetic energy cut off.

## Additional Information

**How to cite this article**: Zhou, T. *et al*. Transition metal ions regulated oxygen evolution reaction performance of Ni-based hydroxides hierarchical nanoarrays. *Sci. Rep.* **7**, 46154; doi: 10.1038/srep46154 (2017).

**Publisher's note:** Springer Nature remains neutral with regard to jurisdictional claims in published maps and institutional affiliations.

## Supplementary Material

Supplementary Information

## Figures and Tables

**Table 1:** OER catalytic performances of the Ni_*y*_M(OH)_*x*_ HNAs and IrO_*x*_ in 1 M KOH.

Electrodes	Onset Potential (mV vs. RHE)	η at *j* = 100 mA cm^−2^ (mV)	*j* at η = 350 mV (mA cm^−2^)	*j*_BET_ at η = 350 mV (mA cm^−2^)	Tafel Slope (mV dec^−1^)	TOF at η = 350 mV (s^−1^)
Ni(OH)_2_	254	423	25.7	0.22	123.4	0.011
Ni_2.2_Fe(OH)_*x*_	234	298	427.9	1.15	64.3	0.165
Ni_2.1_Zn(OH)_*x*_	276	410	25.8	0.13	107.2	0.008
IrO_*x*_	248	375	67.4	—	113.3	0.048
