# Supplementary material for: Transition metal ions regulated oxygen evolution reaction performance of Ni-based hydroxides hierarchical nanoarrays
Source: Sci Rep. 2017 Apr 6;7:46154. doi: 10.1038/srep46154 (PMC5382681; doi:10.1038/srep46154)
Supplement: Supplementary Information [file srep46154-s1.doc]

**Transition metal ions regulated oxygen evolution reaction performance of Ni-based hydroxides hierarchical nanoarrays**

**Tingting Zhou**^1^**, Zhen Cao**^2^**, Pan Zhang**^2^**, Houyi Ma**^1^**, Zhen Gao**^2^**, Heng Wang**^2^**, Yue Lu**^2^**, Jia He**^2*^**, Yunfeng Zhao**^2*^

^1^ School of Chemistry and Chemical Engineering, Shandong University, Jinan 250100, China
^2^ Tianjin Key Laboratory of Advanced Functional Porous Materials, Institute for New Energy Materials and Low-Carbon Technologies, Tianjin University of Technology, Tianjin 300384, China

*Corresponding author.

E-mail address: [hejia@tjut.edu.cn](mailto:hejia@tjut.edu.cn), [yfzhao@tjut.edu.cn](mailto:yfzhao@tjut.edu.cn)

**Table S1.** ICP results of the Ni*_y_*M(OH)_x_ HNAs.

| Number | Sample | Content of Ni (%) | Content of M (%) | Atom ratio ( Ni : M) |
| --- | --- | --- | --- | --- |
| 1 | Ni | 8.73 | 0 | ---- |
| 2 | NiFe | 6.42 | 2.75 | 2.22:1 |
| 3 | NiZn | 8.83 | 4.63 | 2.12:1 |

**
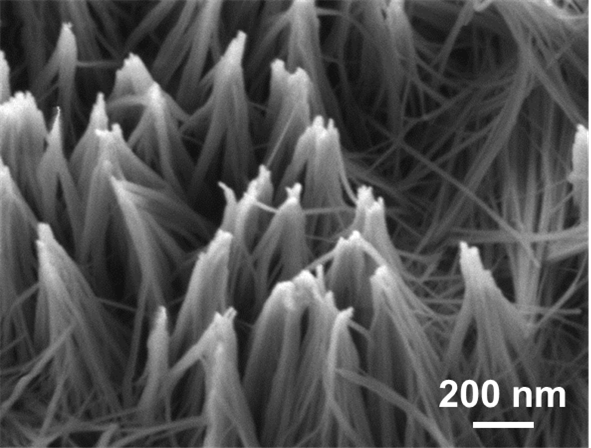
**

**Figure S1.** SEM image of the Cu_2_O nanoarrays on Cu foam.


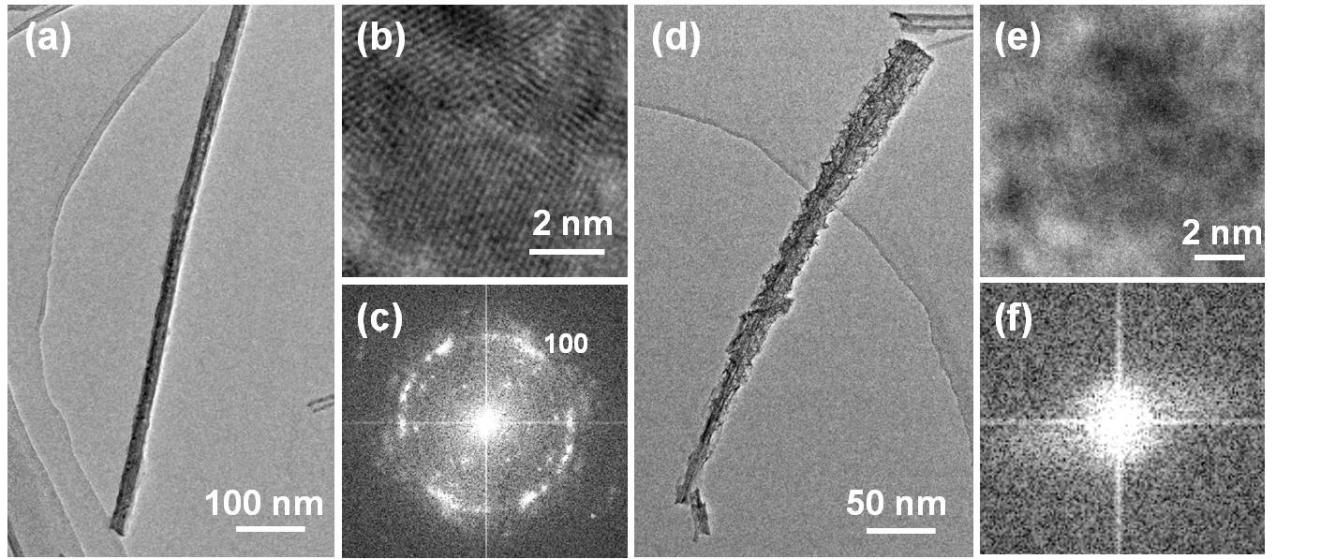


**Figure S2.** (a) Low magnification TEM, (b) HRTEM and (c) FFT images of the Ni(OH)_2_ HNAs; (d) Low magnification TEM, (e) HRTEM and (f) FFT images of the Ni_2.1_Zn(OH)*_x_* HNAs.


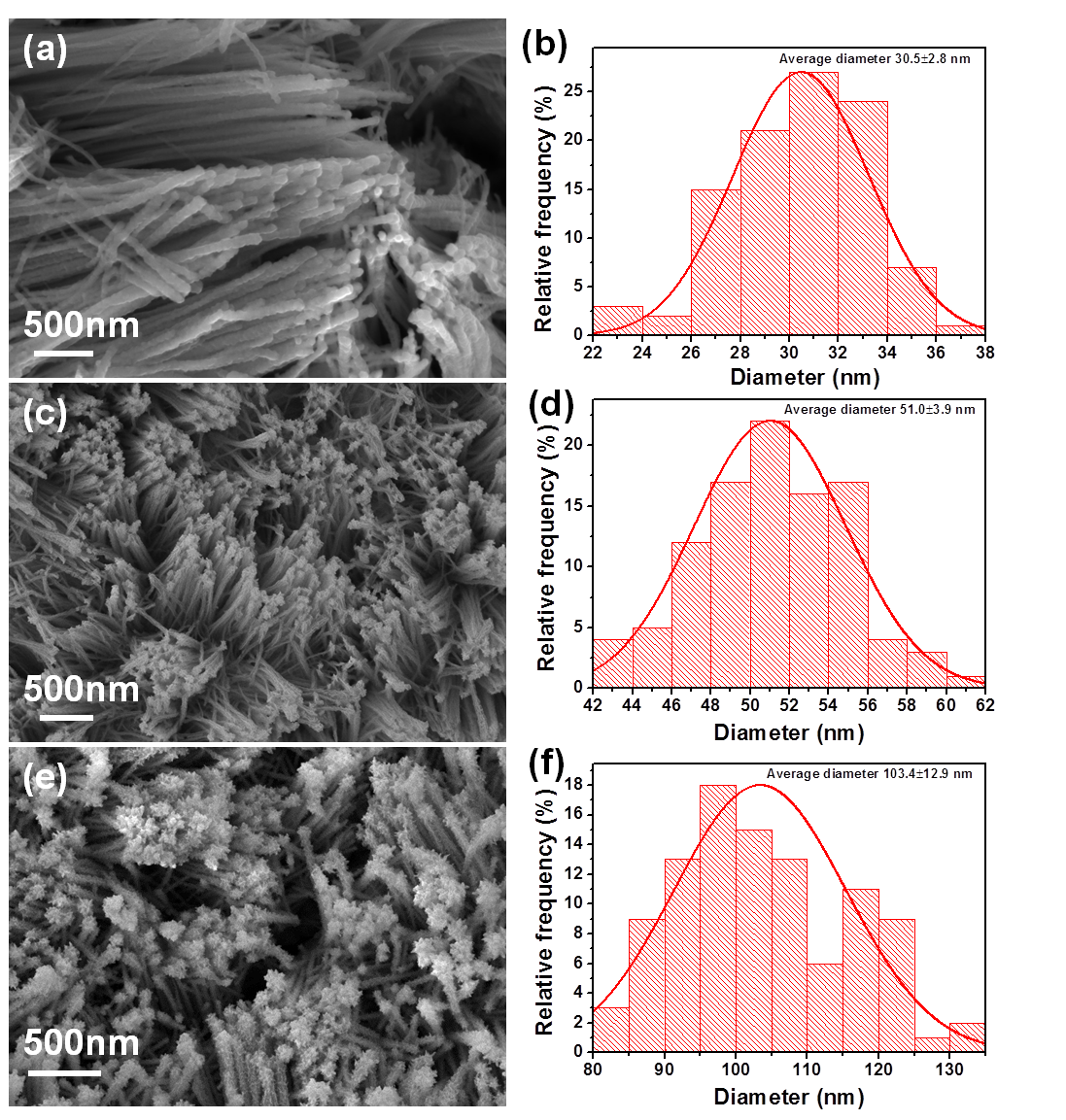


**Figure S3.** (a), (c) and (e) SEM images of the Ni(OH)_2_, Ni_2.1_Zn(OH)*_x_* and Ni_2.2_Fe(OH)*_x_* HNAs, (b), (d) and (f) corresponding diameter distributions along with standard deviations of the Ni(OH)_2_, Ni_2.1_Zn(OH)*_x_* and Ni_2.2_Fe(OH)*_x_* HNAs.


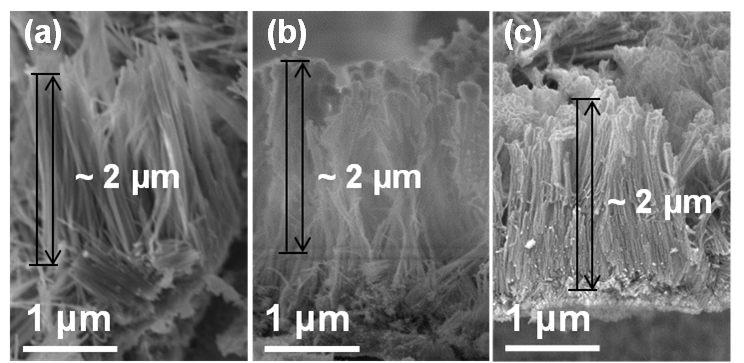


**Figure S4** Cross-sectional SEM images (side view) of the Ni(OH)_2_, Ni_2.1_Zn(OH)*_x_* and Ni_2.2_Fe(OH)*_x_* HNAs.


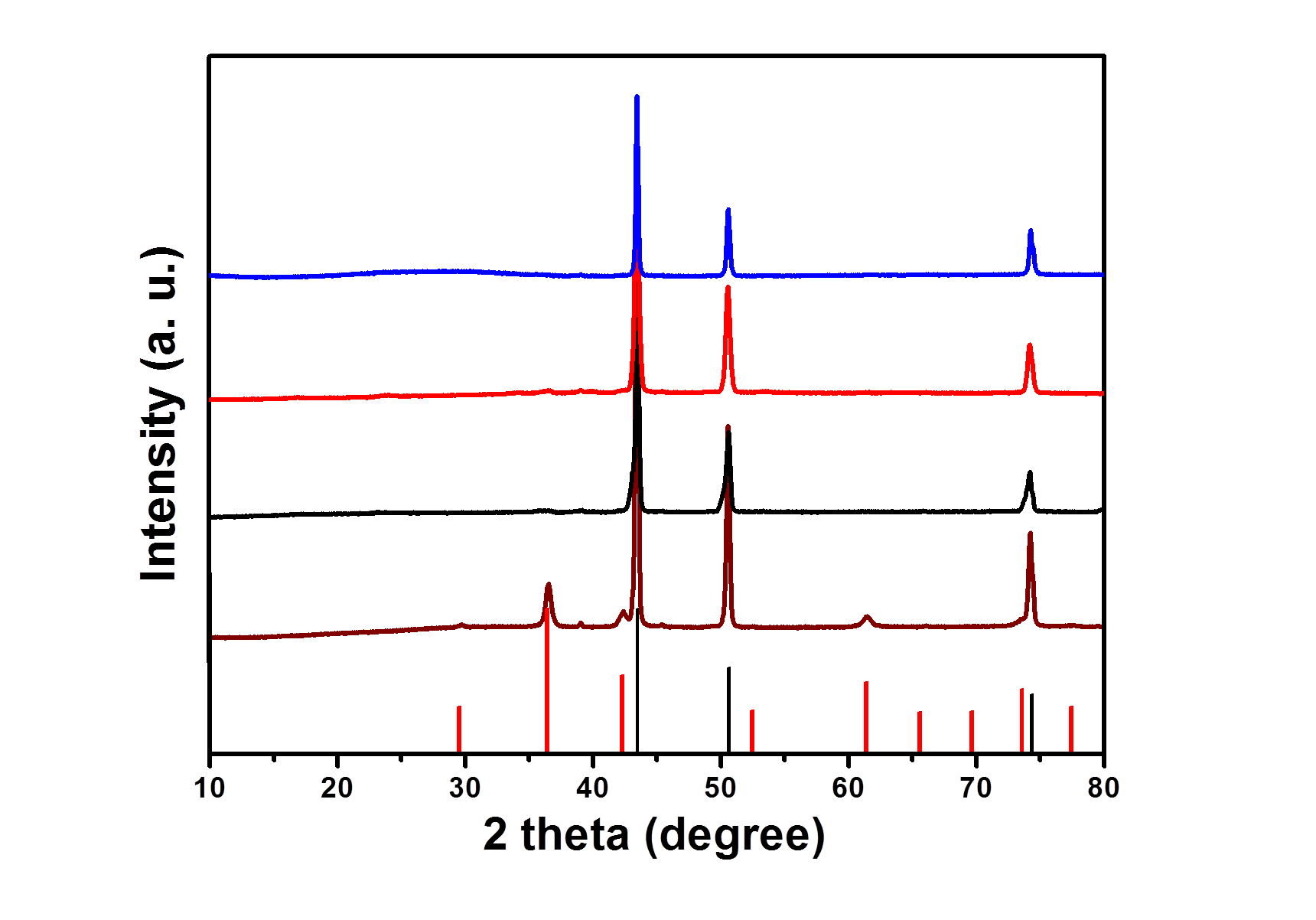


**Figure S5.** XRD patterns for the Cu_2_O template, Ni(OH)_2_, Ni_2.1_Zn(OH)*_x_* and Ni_2.2_Fe(OH)*_x_* HNAs (lines from bottom to top). The peaks are indexed using reference peaks from the appropriate PDF cards: Cu_2_O phases (red, PDF#65-3288) and Cu phases (black, PDF# 65-9743).

**Figure S6.** EIS at η of 300 mV for the Ni*_y_*M(OH)*_x_* HNAs.

**Table S2.** EIS fitting results of the Ni*_y_*M(OH)_x_ HNAs.

| Material | Rs (Ω) | Rct (Ω) | Rcp (Ω) |
| --- | --- | --- | --- |
| **Ni(OH)_2_** | 2.5 | 23.8 | 0.14 |
| **Ni_2.2_Fe (OH)*_x_*** | 2.6 | 1.7 | 0.027 |
| **Ni_2.1_Zn (OH)*_x_*** | 4. 9 | 21.4 | 0.12 |

**
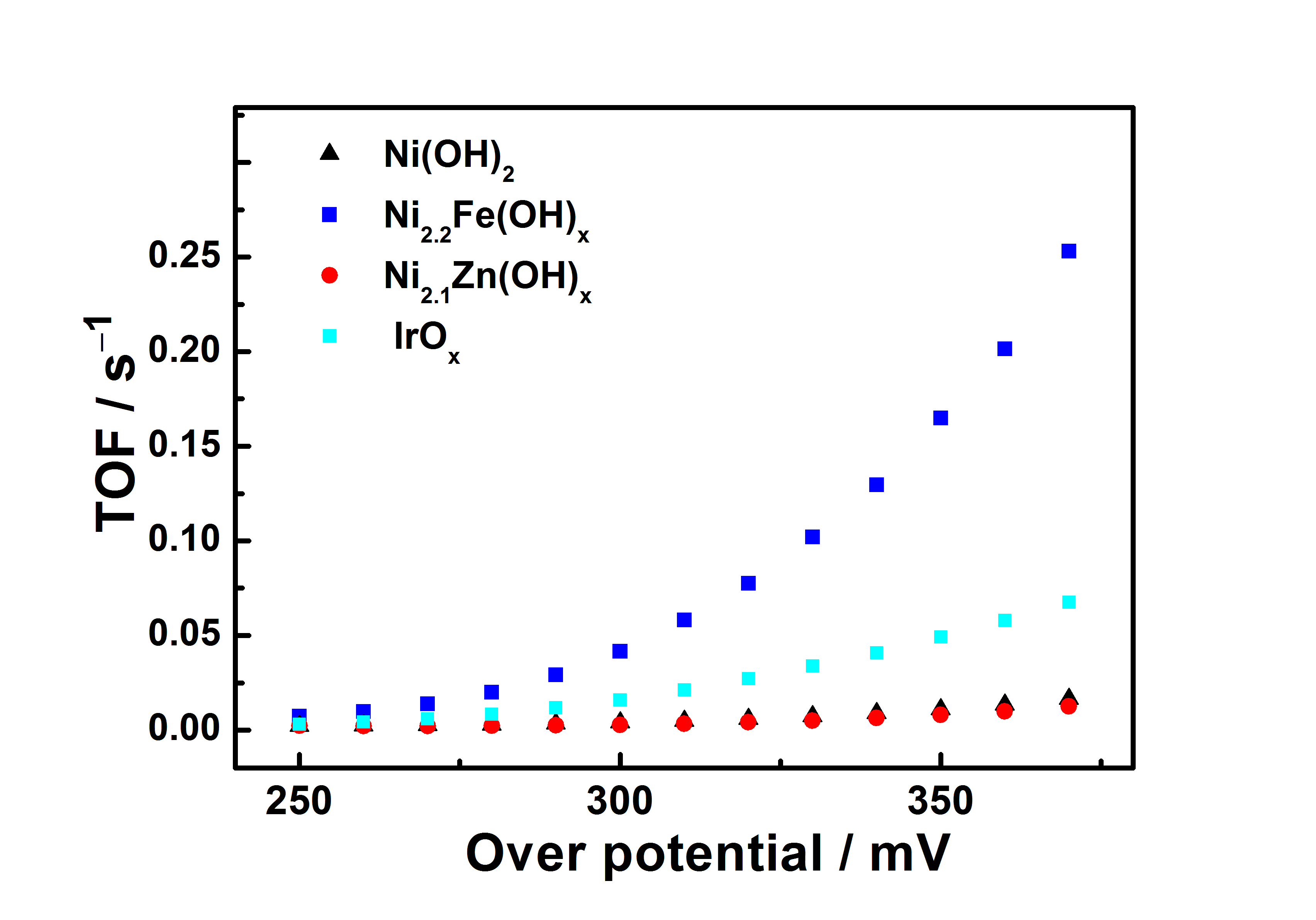
**

**Figure S7** TOF of the Ni*_y_*M(OH)*_x_* HNAs and IrO*_x_*.

**Figure S8** Nitrogen adsorption/desorption isotherms of the Ni*_y_*M(OH)*_x_* HNAs.

**Table S3** The OER performance of typical Co-based electorcatalysts for water oxidation under alkaline solution.

|  | Electrolyte | Onset Poteneial (mV) | η at  10 mA cm^-2^(mV) | Tafel Slope (mV/dec) | Ref. |
| --- | --- | --- | --- | --- | --- |
| NiCo_2_O_4_ core-shell  nanowire | 1 M NaOH | 270 | 320 | 63.1 | [^1^](#_ENREF_1) |
| Zn_x_Co_3−x_O_4_ nanoarrays | 0.1 M KOH | ---- | 320 | 51 | [^2^](#_ENREF_2) |
| Co_3_O_4_ nanocube/CoO | 1 M KOH | ---- | 430 | 89 | [^3^](#_ENREF_3) |
| CoNi(OH)_x_ nanotubes | 1 M KOH | 250 | 280 | 77 | [^4^](#_ENREF_4) |
| CoO*_x_* nanotube | 1 M KOH | 230 | ---- | 75 | [^5^](#_ENREF_5) |
| Hollow NiCo_2_O_4_ | 0.1 M KOH | ---- | 340 | 75 | [^6^](#_ENREF_6) |
| NiCoO_x_ hollow  nanosponges | 0.1 M KOH | 271 | 362 | 73.2 | [^7^](#_ENREF_7) |
| Screw CoNi LDH/C | 1M KOH | 330 | 360 | 38.5 | [^8^](#_ENREF_8) |
| CoFe_2_O_4_ nanoparticles | 1M NaOH | 270 | 378 | 73 | [^9^](#_ENREF_9) |
| Ni–Co oxide nanorods | 1M NaOH | 290 | 350 | 55 | [^10^](#_ENREF_10) |
| NiCo/NF or NiCo/CP | 1 M KOH | ---- | 360 | 50-60 | [^11^](#_ENREF_11) |
| NiCoO_x_ nanoarray | 1 M KOH | 280 | 290 | 79 | [^12^](#_ENREF_12) |
| Co_3_O_4_/C porous  nanowire arrays | 1 M KOH | 240 | 320 | 70 | [^13^](#_ENREF_13) |
| N-doped porous carbon  NSs/CoNi alloy | 0.1 M KOH | 340 | 360 | 165 | [^14^](#_ENREF_14) |
| Co-S nanosheets film | 1 M KOH | 320 | 361 | 64 | [^15^](#_ENREF_15) |
| NiCo hydroxide | 0.1 M KOH | 310 | 460 | 65 | [^16^](#_ENREF_16) |
| CNTs-Au@Co_3_O_4_ | 1 M KOH | 330 | 360 | 68 | [^17^](#_ENREF_17) |
| Hollow fluffy cages Co_3_O_4_ | 1 M KOH | ---- | 409 | 70 | [^18^](#_ENREF_18) |

**References**

1. Chen, R., Wang, H.-Y., Miao, J., Yang, H. & Liu, B. A flexible high-performance oxygen evolution electrode with three-dimensional NiCo_2_O_4_ core-shell nanowires. *Nano Energy* **11**, 333-340 (2015).

2. Liu, X. et al. Hierarchical Zn_x_Co_3–x_O_4_ nanoarrays with high activity for electrocatalytic oxygen evolution. *Chem. Mater.* **26**, 1889-1895 (2014).

3. Bergmann, A. et al. Reversible amorphization and the catalytically active state of crystalline Co3O4 during oxygen evolution. *Nat. Commun.* **6**, 8625 (2015).

4. Li, S. et al. Co-Ni-based nanotubes/nanosheets as efficient water splitting electrocatalysts. *Adv. Energy Mater.* **6**, 1501661 (2016).

5. Wang, Y. et al. Bio-inspired leaf-mimicking nanosheet/nanotube heterostructure as a highly efficient oxygen evolution catalyst. *Adv. Sci.* **2**, 1500003 (2015).

6. Lv, X. et al. Hollow mesoporous NiCo_2_O_4_ nanocages as efficient electrocatalysts for oxygen evolution reaction. *Dalton Trans.* **44**, 4148-4154 (2015).

7. Zhu, C. et al. Nickel cobalt oxide hollow nanosponges as advanced electrocatalysts for the oxygen evolution reaction. *Chem Commun (Camb)* **51**, 7851-7854 (2015).

8. Ni, B. & Wang, X. Edge overgrowth of spiral bimetallic hydroxides ultrathin-nanosheets for water oxidation. *Chem. Sci.* **6**, 3572-3576 (2015).

9. Kargar, A. et al. Solution-processed CoFe_2_O_4_ nanoparticles on 3D carbon fiber papers for durable oxygen evolution reaction. *ACS Appl. Mater. Inter.* **7**, 17851-17856 (2015).

10. Wang, H.-Y. et al. Ni^3+^-induced formation of active NiOOH on the spinel Ni-Co oxide surface for efficient oxygen evolution reaction. *Adv. Energy Mater.* **5**, 1500091 (2015).

11. Xiao, Y. et al. NiCo_2_O_4_ 3 dimensional nanosheet as effective and robust catalyst for oxygen evolution reaction. *RSC Adv.* **5**, 61900-61905 (2015).

12. Lu, Z. et al. Superaerophobic electrodes for direct hydrazine fuel cells. *Adv. Mater.* **27**, 2361-2366 (2015).

13. Ma, T., Dai, S., Jaroniec, M. & Qiao, S. Metal-organic framework derived hybrid Co_3_O_4_-carbon porous nanowire arrays as reversible oxygen evolution electrodes. *J. Am. Chem. Soc.* **136**, 13925-13931 (2014).

14. Hou, Y. et al. Strongly coupled 3D hybrids of N-doped porous carbon nanosheet/CoNi alloy-encapsulated carbon nanotubes for enhanced electrocatalysis. *Small* **11**, 5940-5948 (2015).

15. Liu, T. et al. Electrodeposition of cobalt-sulfide nanosheets film as an efficient electrocatalyst for oxygen evolution reaction. *Electrochem. Commun.* **60**, 92-96 (2015).

16. Zhao, Z., Wu, H., He, H., Xu, X. & Jin, Y. A high-performance binary Ni-Co hydroxide-based water oxidation electrode with three-dimensional coaxial nanotube array structure. *Adv. Funct. Mater.* **24**, 4698-4705 (2014).

17. Fang, Y. et al. Ultrasonication-assisted ultrafast preparation of multiwalled carbon nanotubes/Au/Co_3_O_4_ tubular hybrids as superior anode materials for oxygen evolution reaction. *J. Power Sources* **300**, 285-293 (2015).

18. Zhou, X. et al. Hollow fluffy Co_3_O_4_ cages as efficient electroactive materials for supercapacitors and oxygen evolution reaction. *ACS Appl. Mater. Inter.* **7**, 20322-20331 (2015).
